# A Biomechanical Simulation of Forearm Flexion Using the Finite Element Approach

**DOI:** 10.3390/bioengineering11010023

**Published:** 2023-12-25

**Authors:** Chenyang Liang, Fei Jiang, Daisuke Kawaguchi, Xian Chen

**Affiliations:** Department of Mechanical Engineering, Graduate School of Sciences and Technology for Innovation, Yamaguchi University, Tokiwadai, Ube 7558611, Yamaguchi, Japan; c505wdw@yamaguchi-u.ac.jp (C.L.); b026vd@yamaguchi-u.ac.jp (D.K.); xchen@yamaguchi-u.ac.jp (X.C.)

**Keywords:** upper limb, forearm flexion, muscle contraction, Hill-type transversely isotropic hyperelastic material, finite element method

## Abstract

Upper limb movement is vital in daily life. A biomechanical simulation of the forearm with consideration of the physiological characteristics of the muscles is instrumental in gaining deeper insights into the upper limb motion mechanisms. In this study, we established a finite element model of the forearm, including the radius, biceps brachii, and tendons. We simulated the motion of the forearm resulting from the contraction of the biceps brachii by using a Hill-type transversely isotropic hyperelastic muscle model. We adjusted the contraction velocity of the biceps brachii muscle in the simulation and found that a slower muscle contraction velocity facilitated forearm flexion. Then, we changed the percentage of fast-twitch fibers, the maximum muscle strength, and the neural excitation values of the biceps brachii muscle to investigate the forearm flexion of elderly individuals. Our results indicated that reduced fast-twitch fiber percentage, maximum muscle strength, and neural excitation contributed to the decline in forearm motion capability in elderly individuals. Additionally, there is a threshold for neural excitation, below which, motion capability sharply declines. Our model aids in understanding the role of the biceps brachii in forearm flexion and identifying the causes of upper limb movement disorders, which is able to provide guidance for enhancing upper limb performance.

## 1. Introduction

Upper limb movement is an essential part of daily life. Biomechanical simulations of the forearm, incorporating the salient physiological attributes of the upper limb muscles, serve as a pivotal tool for acquiring profound insights into the mechanisms governing upper limb motion. Many researchers have employed various methods for biomechanical simulations related to the upper limb. Ambrosio et al. [1] developed a multibody biomechanical model of the upper limb and conducted simulations to predict the muscle forces during humeral elevation. Teran et al. [2] developed a comprehensive upper limb model using finite volume methods and the finite element method, and conducted an inverse dynamics analysis. However, they adopted a simple constitutive model for muscle without consideration of the force-velocity relationship. Dereshgi et al. (2022) [3] investigated the mechanical behaviors of muscle fibers during dumbbell curl exercises with varying dumbbell weights. Later, they investigated the rest-pause biceps curl exercise effect on the biceps brachii muscles of women [4]. However, they simplified the upper limb model into basic geometric shapes, failing to accurately depict the true structural characteristics of the upper limb. Chen et al. [5] constructed a realistic upper limb model from MRI images and simulated forearm movements using the finite element method. However, the accuracy of the results was low due to the small number of elements used in their simulation. Blemker et al. [6] utilized the finite element method to simulate the contraction of the biceps brachii muscle and investigated the nonuniform shortening behavior of the biceps brachii muscle.

The finite element method is a common approach for simulating upper limb movements. In finite element modeling, the paramount component governing the kinetics of forearm flexion resides in the biceps brachii muscles. In general, muscular contraction is initiated through the cerebral transmission of neural excitation to the muscle fibers. [7,8]. After muscle contraction, tendons transmit tension to the bones, enabling joint movement and the motion of flexion. Muscles exhibit complex mechanical behavior, and they are usually regarded as incompressible transversely isotropic hyperelastic materials [9]. Additionally, there is a strain rate effect on the mechanical response of the muscle tissue [10,11]. The modeling of the biomechanical behavior of muscles is the key point for the finite element simulation, which includes both passive and active behavior. Passive behavior pertains to the intrinsic elasticity and compliance of both muscle fibers and the isotropic matrix. These properties enable muscles to undergo elongation and subsequently revert to their initial length in response to externally applied forces. This passive behavior can be delineated by nonlinear hyperelasticity, which elucidates the connection between muscle forces and the degree of stretching. On the other hand, active behavior represents the contractile characteristics of muscle fibers driven by neural excitation. The muscle forces generated by active behavior are associated with muscle contraction velocity and neural excitation level. The biomechanical behavior of muscles encompasses dynamic interactions between passive and active components. Therefore, the muscle force is a function of contraction velocity, neural excitation from the active part and stretch ratio from the passive part. The mathematical modeling of muscles needs to carefully consider these factors [12].

The Hill-type muscle model integrates both active and passive behaviors of muscles, and it is widely applied in biomechanical research [12,13]. However, the original Hill-type model is one-dimensional. Its applicability to real muscles, characterized by intricate three-dimensional (3D) structures, is limited. Many researchers have extended the one-dimensional Hill-type model to three dimensions to simulate the mechanical properties of various muscle tissues with real geometries [13,14,15,16,17,18]. Kojic et al. [14] proposed a 3D Hill-type model in which passive elastic behavior was simplified to be 3D linear elastic and isotropic, without considering the hyperelastic behavior of muscles. Furthermore, they did not consider the incompressibility of muscle tissue. Tang et al. [15] used this model to simulate the mechanical behavior of skeletal muscles considering fatigue factors. In contrast, Johansson et al. [16] proposed a 3D Hill-type model that considered the incompressibility of muscles. However, the passive behaviors of muscle fibers and the matrix that embeds these fibers were conflated in this model. Martins et al. [17] introduced an incompressible, transversely isotropic hyperelastic 3D muscle model that included both active and passive muscle behavior. However, this model did not consider the strain rate effects of muscles. Subsequently, Martins et al. [18] improved this model by incorporating the effects of strain rate on muscles. This muscle model appeared to simulate muscle contraction under real conditions. However, muscle fiber types, in terms of fast-twitch and slow-twitch fibers, have not been considered yet in this model. Research about the effect of muscle fiber composition on motion ability remains limited. Additionally, sarcopenia, defined as the loss of muscle mass and strength with age, is recognized as a major cause of disability and morbidity in elderly individuals, such as IADL (instrumental activities of daily living) disability [19]. Sarcopenia results from a combination of changes in muscle fiber composition, reduced muscle strength associated with muscle atrophy, and neurological dysfunction [20,21]. These factors have not been quantitatively investigated. The objective of this study was to extend the improved Hill-type muscle model [18] and employ the finite element method to simulate the effect of sarcopenia on forearm flexion movements.

## 2. Materials and Methods

### 2.1. Forearm Finite Element Model

**Figure 1:**
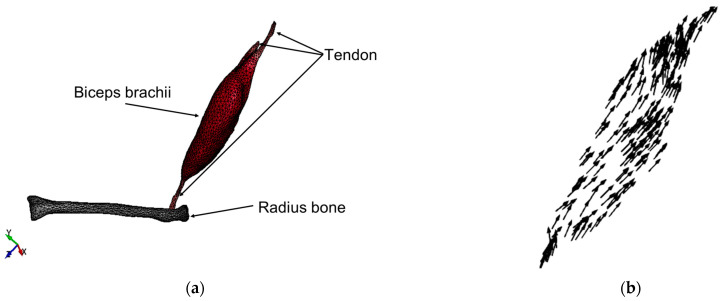
Finite element model of the forearm: (**a**) tetrahedral mesh elements of the model and (**b**) muscle fiber direction of the biceps brachii.

We utilized CT images and the image processing software ScanIP (simpleware ScanIP, version M-2019.06; Synopsys Inc., Mountain View, CA, USA) to construct a 3D model of the forearm, which included the biceps brachii, tendon, and radius bone. The radius bone was automatically segmented using a single threshold segmentation method, while the biceps brachii muscle and tendon were manually segmented. Subsequently, a smoothing procedure was applied to the entire forearm model. Finally, we used the meshing software HyperMesh (Altair HyperMesh, version 2019.1.2; Altair Engineering Inc., Troy, MI, USA) to create a mesh for the forearm model (Figure 1a). The forearm finite element model comprises 16,001 tetrahedral elements and 4580 nodes. In this study, we considered a simplified model only composed of the radius bone, the biceps brachii, and tendon, which is enough to reproduce the forearm flexion motion.

To reproduce the contraction motion of the biceps brachii muscle, the muscle fibers’ direction information is necessary. As described later, the active contractile force will be generated along the fibers’ direction. Therefore, we solved a Laplace’s equation to determine the direction of the biceps brachii muscle fibers. The direction of the resulting gradient is assumed to be able to mimic the direction of the muscle fibers (Figure 1b). The obtained fiber directions (Figure 1b) exhibit a reasonable concordance with the anatomical literature [22].

### 2.2. Constitutive Models of the Muscle, Bone, and Tendon

The muscle was modeled as a fiber-reinforced composite in which the strain energy density function includes the contribution from the isotropic matrix embedding the muscle fiber and the contribution from the muscle fibers [23]. The strain energy density function of the muscle contains the following terms:

(1) $$U=U_{V}+U_{I}+U_{f}$$

where $U_{I}$ represents the strain energy in the isotropic matrix embedding the muscle fiber and $U_{f}$ represents the strain energy of the muscle fiber. $U_{V}$ means the strain energy related to the volume change, given as:

(2) $$U_{V}=2(J-1)$$

where J represents the determinant of the deformation gradient F (J=detF).

As for $U_{I}$, we used the constitutive model proposed by Humphrey et al. [24]:

(3) $$U_{I}={cexp}^{b\left({\tilde{I}}_{C}-3\right)}-1$$

where ${\tilde{I}}_{C}\;$is the first reducing invariant of the right Cauchy–Green deformation tensor C, defined as ${\tilde{I}}_{C}=J^{-2/3}trC$. The right Cauchy–Green deformation tensor C is defined as C=2E+I, where ***E*** and I are the Green–Lagrange deformation tensor and the identity tensor, respectively. c and b are the constants of this model. In this study, we used $c=3.795\times 10^{-4}$ Mpa and b=23.46 [24].

**Figure 2:**
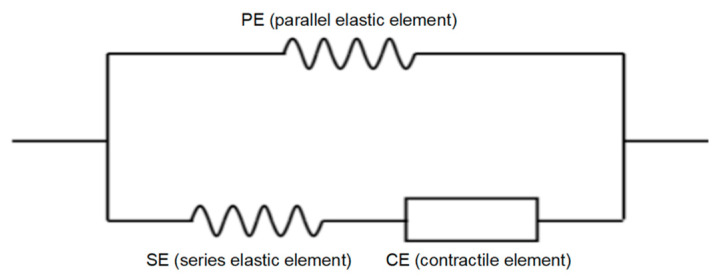
Hill-type three-element model of the muscle.

As for $U_{f}$, we employed the skeletal muscle fiber strain energy function proposed by Martins et al. [18]. This function utilizes the Hill-type three-element model (Figure 2), incorporating a realistic representation of muscle mechanics. The Hill-type model comprises three core components: a Contractile Element (CE), a Series Element (SE), and a Parallel Element (PE). The CE is responsible for generating contractile force during muscle activation and does not contribute to passive forces when subjected to passive stretching. It is typically recognized as the sliding actin and myosin filaments. The SE, linked in series with the CE, behaves as a non-linear spring and is attributed to the inherent elasticity found within the myofilaments and cross-bridges. The PE, connected in parallel with the CE, functions as another non-linear spring. It is associated with the elasticity of connective tissues (epimysium, perimysium, and endomysium), as well as the sarcolemma.

The total nominal stress T along the muscle fiber direction can be considered as the sum of the nominal stress produced by the PE and either the CE or SE elements.

(4) $$T=T^{PE}+T^{SE}=T^{PE}+T^{CE}$$

The stress $T^{PE}$ related to the PE elements is defined as follows:

(5) $$T^{PE}=T_{0}^{M}f_{PE}({\tilde{\lambda }}_{f})$$

(6) $$f_{PE}\left({\tilde{\lambda }}_{f}\right)=\left\{\begin{array}{ll} 2aA\exp\left[a{\left({\tilde{\lambda }}_{f}-1\right)}^{2}\right]\left({\tilde{\lambda }}_{f}-1\right) & {\tilde{\lambda }}_{f}>1.0\\ 0 & other \end{array}\right.$$

where ${\tilde{\lambda }}_{f}\;$is the stretch ratio in the direction of the muscle fibers, which is calculated by ${\tilde{\lambda }}_{f}={\left[J^{-2/3}\;C:\left(N_{m}⊗N_{m}\right)\right]}^{1/2}$. $N_{m}$ is the initial direction of the muscle fibers. $T_{o}^{M}$ is the maximum nominal stress of the muscle fibers, using the values $T_{o}^{M}=0.535$ Mpa. A and a are material constants. In this study, we used the values $A=8.568\times 10^{-4}$ and a=12.43 [18].

The stress $\;T^{CE}$ of the CE element comprises three components: (1) the stretch-ratio-related part $f_{L}^{CE}$, which is a function of the stretch ratio of the muscle fibers ${\tilde{\lambda }}_{f}$, (2) the contraction-velocity-related part $f_{V}^{CE}$, which is a function of the contraction velocity of the muscle fibers ${\dot{\tilde{\lambda }}}_{f}$, and (3) the activation level α, which is a function of the time t:

(7) $$T^{CE}\left({\tilde{\lambda }}_{f},{\dot{\tilde{\lambda }}}_{f},\alpha (t)\right)=T_{o}^{M}f_{L}^{CE}\left({\tilde{\lambda }}_{f}\right)f_{V}^{CE}\left({\dot{\tilde{\lambda }}}_{f}\right)\alpha (t)$$

The stretch-ratio-related function $f_{L}^{CE}$ and the contraction-velocity-related function $f_{V}^{CE}$ are defined as follows:

(8) $$f_{L}^{CE}\left({\tilde{\lambda }}_{f}\right)=\left\{\begin{array}{ll} -4{\left({\tilde{\lambda }}_{f}-1\right)}^{2}+1 & 0.5\leq {\tilde{\lambda }}_{f}\leq 1.5\\ 0 & other \end{array}\right.$$

(9) $$f_{V}^{CE}\left({\dot{\tilde{\lambda }}}_{f}\right)=\left\{\begin{array}{ll} 0 & {\dot{\tilde{\lambda }}}_{f}\leq -10s^{-1}\\ -\frac{1}{atan(5)}\mathrm{atan}\left(-0.5{\dot{\tilde{\lambda }}}_{f}\right)+1 & -10s^{-1}\leq {\dot{\tilde{\lambda }}}_{f}\leq 2s^{-1}\\ \frac{\pi }{4atan(5)}+1 & {\dot{\tilde{\lambda }}}_{f}>2s^{-1} \end{array}\right.$$

where ${\dot{\tilde{\lambda }}}_{f}>0$ during muscle fiber lengthening and ${\dot{\tilde{\lambda }}}_{f}<0$ during muscle fiber contraction. The absolute value of ${\dot{\tilde{\lambda }}}_{f}$ represents the rate of muscle fiber lengthening or contraction. The activation level α controls the muscle contraction intensity. Our study adopted the same activation formula as Pandy et al. [25], and its expression is as follows:

(10) $$\alpha =\frac{\frac{u}{\tau_{\mathrm{rise}\;}}+\frac{1}{\tau_{\mathrm{fall}\;}}\alpha_{\min\;}(1-u)}{\frac{u}{\tau_{\mathrm{rise}\;}}+\frac{1}{\tau_{\mathrm{fall}\;}}(1-u)}\cdot \left(1-e^{-t\left[\frac{u}{\tau_{\mathrm{rise}\;}}+\frac{1}{\tau_{\mathrm{fall}\;}}(1-u)\right]}\right)$$

where $\tau_{\mathrm{rise}\;}$ and $\tau_{\mathrm{fall}\;}$ represent the characteristic time constants governing the activation and deactivation processes of the muscle, respectively. They determine how quickly the muscle reaches its peak activation level and how rapidly it returns to its resting state after activation. The experimental values $\tau_{\mathrm{rise}\;}=0.02$ and $\tau_{\mathrm{fall}\;}=0.2$ were provided by Pandy et al. [25]. u represents the neural excitation, taking values from 0 to 0.5.

The strain energy produced by the muscle fibers can be written as follows:

(11) $$U_{f}\left({\tilde{\lambda }}_{f},{\dot{\tilde{\lambda }}}_{f},\alpha \right)=U_{PE}\left({\tilde{\lambda }}_{f}\right)+U_{CE}\left({\tilde{\lambda }}_{f},{\dot{\tilde{\lambda }}}_{f},\alpha \right)$$

where,

(12) $$U_{PE}\left({\tilde{\lambda }}_{f}\right)=T_{o}^{M}\int_{1}^{{\tilde{\lambda }}_{f}}f_{PE}\left({\tilde{\lambda }}_{f}\right)d{\tilde{\lambda }}_{f}$$

(13) $$U_{CE}\left({\tilde{\lambda }}_{f},{\dot{\tilde{\lambda }}}_{f},\alpha \right)=\alpha \cdot T_{o}^{M}\int_{1}^{{\tilde{\lambda }}_{f}}\;f_{L}^{CE}\left({\tilde{\lambda }}_{f}\right)f_{V}^{CE}\left({\dot{\tilde{\lambda }}}_{f}\right)d{\tilde{\lambda }}_{f}$$

In summary, the biceps brachii muscle can be regarded as an incompressible, transversely isotropic, hyperelastic material which is assumed to be governed by a constitutive law linking the second Piola–Kirchhoff stress tensor S and the Green–Lagrange deformation tensor E. The final second Piola–Kirchhoff stress tensor of the biceps brachii muscle is as follows:

(14) $$\begin{array}{ll} S & =\frac{\partial U}{\partial E}=\frac{\partial U_{J}}{\partial E}+\frac{\partial U_{I}}{\partial E}+\frac{\partial U_{f}}{\partial E}=\frac{\partial U_{J}}{\partial J}\frac{\partial J}{\partial E}+\frac{\partial U_{I}}{\partial {\tilde{I}}_{C}}\frac{\partial {\tilde{I}}_{C}}{\partial E}+\frac{\partial U_{f}}{\partial {\tilde{\lambda }}_{f}}\frac{\partial {\tilde{\lambda }}_{f}}{\partial E}\\ & ={2JC}^{-1}+\left\{bce^{b\left({\tilde{I}}_{C}-3\right)}\right\}\left(2J^{-3/2}I-\frac{2}{3}{\tilde{I}}_{C}C^{-1}\right)+\frac{\partial U_{f}}{\partial {\tilde{\lambda }}_{f}}\left(J^{-2/3}{\tilde{\lambda }}_{f}^{-1}(N_{m}⊗N_{m})-\frac{1}{3}{\tilde{\lambda }}_{f}C^{-1}\right) \end{array}$$

Furthermore, the radius bone and the tendon were treated as isotropic elastic materials, and the constitutive equation can be written as:

(15) $$S=\lambda_{m}tr\left(E\right)+2\mu E$$

where tr means the trace. The Lame parameters μ  and $\lambda_{m}$ were 6154 and 9231 MPa, respectively, for the radius bone, and 56 and 9296 MPa, respectively, for the tendon [26,27].

### 2.3. Finite Element Discretization

Skeletal muscles are typically regarded as incompressible materials [13]. We employed the Lagrange multiplier method to constrain the volumetric changes in the muscle. The incompressibility condition was treated by introducing a constraint function $W=J^{2}-1$. The total potential energy of the muscle can be expressed as a function concerning the displacement u and the Lagrange multiplier λ:

(16) $$\Phi \left(u,\lambda \right)=\int_{\Omega }U\mathrm{d}\Omega +\int_{\Omega }\lambda Wd\Omega -\int_{\Gamma }t\cdot udS$$

where Ω and Γ indicate the volume and boundary surface, respectively. t represents the external conservative force on the boundary. The Lagrange multiplier λ is responsible for enforcing the incompressible constraint. According to the principle of minimum potential energy, the variation in the total potential energy Φ is equal to zero:

(17) $$\delta \Phi =\int_{\Omega }\frac{\partial U}{\partial E}\delta Ed\Omega +\int_{\Omega }\left(\lambda \frac{\partial W}{\partial E}\delta E+\delta \lambda W\right)d\Omega -\int_{\Gamma }t\cdot \delta udS=0$$

The necessary condition for the satisfaction of Equation (17) with arbitrary δu and δλ is:

(18) $$\left\{\begin{array}{l} \int_{\Omega }\delta E:S_{inc}\mathrm{d}\Omega =\int_{\Gamma }t\cdot \delta u\mathrm{d}S\;\;\;\;\\ \int_{\Omega }\delta \lambda Wd\Omega =0\;\;\;\;\;\;\;\;\;\;\;\;\;\;\;\;\;\;\;\;\;\;\;\;\;\;\;\;\; \end{array}\right.$$

where the second Piola–Kirchhoff stress tensor under incompressible conditions can be written as:

(19) $$S_{inc}=\frac{\partial U}{\partial E}+\lambda \frac{\partial W}{\partial E}$$

The Galerkin procedure was adopted for spatial discretization. The shape function matrix for displacement u and virtual displacement δu is denoted as N. Similarly, the shape function matrix for Lagrange multiplier λ and virtual Lagrange multiplier δλ is denoted as M. The discretization of u, δu, λ, and δλ within each element is as follows:

(20) $$u=Nu^{\left(n\right)}\;\;\;\;\;\;\;\;\;\;\delta u=N\delta u^{\left(n\right)}$$

(21) $$\lambda =M\lambda^{\left(m\right)}\;\;\;\;\;\;\;\;\;\;\delta \lambda =M\delta \lambda^{\left(m\right)}$$

where n and m are the number of nodes and the number of the Lagrange multipliers within each element, respectively. The final discretized equation can be expressed as follows:

(22) $$\left\{\begin{array}{l} {\left\{\delta u\right\}}^{T}\int_{\Omega }{\left[B\right]}^{T}\left\{S_{inc}\right\}d\Omega ={\left\{\delta u\right\}}^{T}\int_{\Gamma }{\left[N\right]}^{T}\left\{t\right\}dS\;\;\;\;\;\;\;\;\;\\ {\left\{\delta \lambda \right\}}^{T}\int_{\Omega }{\left[M\right]}^{T}Wd\Omega =0\;\;\;\;\;\;\;\;\;\;\;\;\;\;\;\;\;\;\;\;\;\;\;\;\;\;\;\;\;\;\;\;\;\;\;\;\;\;\;\;\;\;\;\;\;\;\; \end{array}\right.$$

where [B] is the strain–displacement matrix for a large deformation problem [28].

Our finite element model was validated by performing uniaxial passive stretching and active contraction simulations. More details can be referred to in Appendix A.

## 3. Results and Discussion

### 3.1. The Effect of Biceps Brachii Contraction Velocity on Forearm Flexion

**Figure 3:**
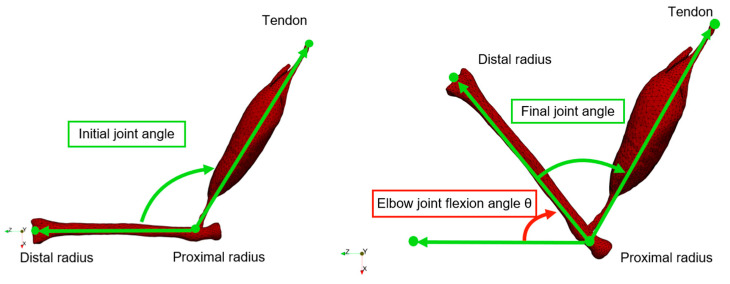
The definition of elbow joint flexion angle.

Analyzing the elbow joint flexion angle under certain load condition offers valuable insights into the forearm’s mobility and muscle performance [29,30]. Our developed finite element model can reproduce the forearm flexion motion (Video S1). The elbow joint flexion angle is defined as the difference between the final joint angle (right side of Figure 3) and the initial joint angle (left side of Figure 3).

**Figure 4:**
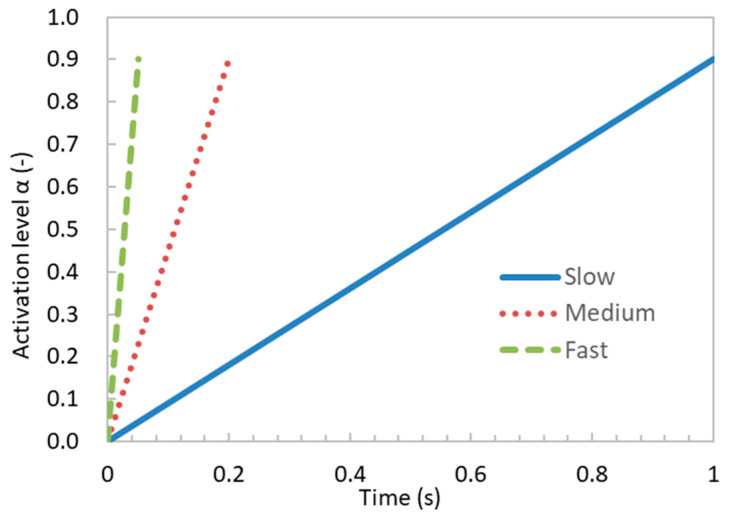
Temporal variations in muscle activation level with different increasing rates (fast, medium, and slow). The uniformity of the maximum activation level was maintained for each case ($\alpha_{max}=0.91$).

**Figure 5:**
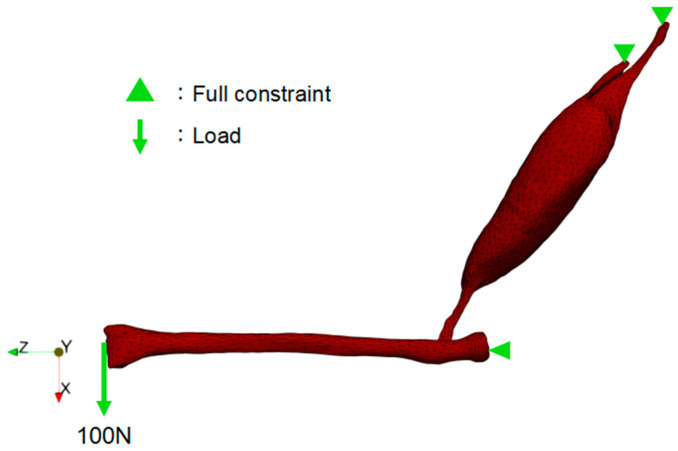
Forearm simulation boundary conditions: complete constraints were imposed on the nodes located at the distal extremities of the biceps brachii tendon heads, as well as the single node at the proximal end of the radius; displacement along the *Y*-axis was restricted at both ends of the radius; a vertical downward force, with a maximum magnitude of 100 N, was applied at the anterior end of the radius.

The maximum contraction velocity of muscles is related to their activation level [31]. A more rapid increase in muscle activation level results in a quicker muscle contraction velocity. Therefore, we controlled the muscle’s contraction velocity by changing the activation level increase rates (Figure 4). We simulated the impacts of the contraction velocities (fast, medium, and slow) on the forearm flexion by using the linearly increasing activation level with different rates, as shown in Figure 4. The temporal duration for attaining the pinnacle of activation, denoted as $\alpha_{max}$, within the fast, medium, and slow cases, amounted to 0.05 s, 0.20 s, and 1.00 s, correspondingly. For the boundary conditions, we imposed full constraints on the long head of the biceps brachii tendon and the proximal end of the radius. To restrict the supination of the forearm, we also constrained the *Y*-axis displacement of the nodes near both ends of the radius. Additionally, a vertically downward load was applied at the anterior end of the radius (Figure 5). This load was gradually applied from zero to the maximum value (100 N) along with the increase in the activation level.

**Figure 6:**
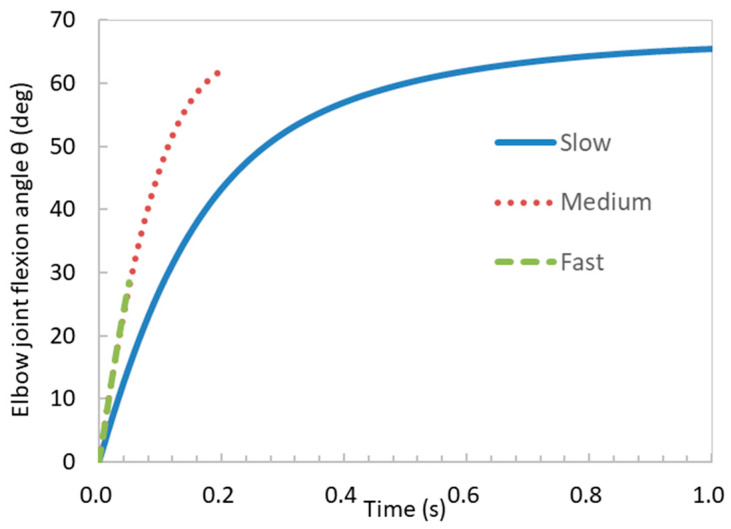
Temporal variations in elbow joint flexion angle for different muscle contraction velocities.

**Figure 7:**
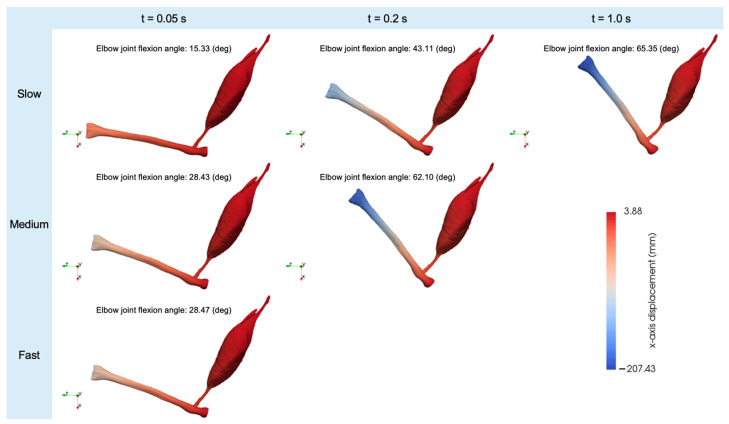
The displacements of the forearm along the *x*-axis and the elbow joint flexion angles at various times (fast: t = 0.05 s, medium: t = 0.05 s and t = 0.2 s, and slow: t = 0.05 s, t = 0.2 s, and t = 1.0 s).

The results showed that the elbow joint flexion angle increased over time until the activation level reached its maximum value in all cases (Figure 6 and Figure 7). In the medium and slow cases, the rate of increase in the elbow joint flexion angle gradually decreased over time. The highest degrees of elbow joint flexion achieved in the fast, medium, and slow cases were recorded as 28.47, 62.10, and 65.35 degrees, respectively. The force–velocity (F-V) curve of the skeletal muscles during concentric contraction demonstrates that muscle force decreases with an increasing contraction velocity [32]. This decrease in muscle force leads to a reduction in the elbow joint flexion angle. In other words, an increase in the contraction velocity leads to a diminishing maximum elbow joint flexion angle. Our results agreed with the F-V curve.

From these results, we observed that larger contraction velocities of the biceps brachii muscle are less advantageous for promoting forearm flexion. When ${\dot{\tilde{\lambda }}}_{f}<-10$, the strain energy produced by the CE element remains constant at zero (Equations (9) and (13)), leading to diminished contraction stress (Equation (14)) of the muscle fibers, ultimately resulting in a reduction in the elbow joint flexion angle. According to Alcazar et al. [33], standard subjects exhibit a negative relationship between muscle force and contraction velocity during forearm flexion. When the velocity of muscle contraction surpasses a critical threshold, the muscle becomes incapable of exerting substantial force under a particular load [33]. Our simulation results matched with this previous finding. Therefore, we confirmed that our forearm model can reasonably reproduce the forearm’s flexion movement.

### 3.2. Simulation of Forearm Flexion Movement in Elderly Individuals

Sarcopenia (muscle loss with aging) is a common disease in elderly individuals, leading to functional decline, disability, frailty, and falls. Changes in muscle fiber composition, muscle strength decline, and neurological dysfunction are the characteristics of sarcopenia [21,34,35]. By adjusting the relevant parameters of the biceps brachii model, we quantitatively investigated the influences of sarcopenia on functional decline by simulating the forearm’s flexion movement: (1) changing the composition of the muscle fibers, i.e., the percentage of fast-twitch muscle fibers (refer to Section 3.2.1); (2) adjusting the maximum nominal stress $T_{o}^{M}$ to reproduce muscle strength decline; and (3) modifying the neural excitation u to mimic neurological dysfunction. We further conducted a comparative analysis of the influence of these parameters on the forearm flexion motion. All simulation boundary conditions remained consistent with those detailed in the preceding section, except that the load (100 N) was constantly applied throughout the simulation period, mimicking the condition of the dumbbell curl exercise.

#### 3.2.1. Changes in Muscle Fiber Composition

Muscles consist of two primary types of fibers: fast-twitch (Type II) and slow-twitch (Type I) fibers. Slow-twitch fibers contract slowly and are fatigue-resistant, making them well-suited for endurance activities such as long-distance running or maintaining posture. Conversely, fast-twitch fibers contract rapidly but fatigue more quickly. The number of fast-twitch motor units have been found to decrease with human aging [36]. The proportion of slow-twitch and fast-twitch muscle fibers exhibits interindividual variability and can be influenced by factors such as genetics and training [37].

**Figure 8:**
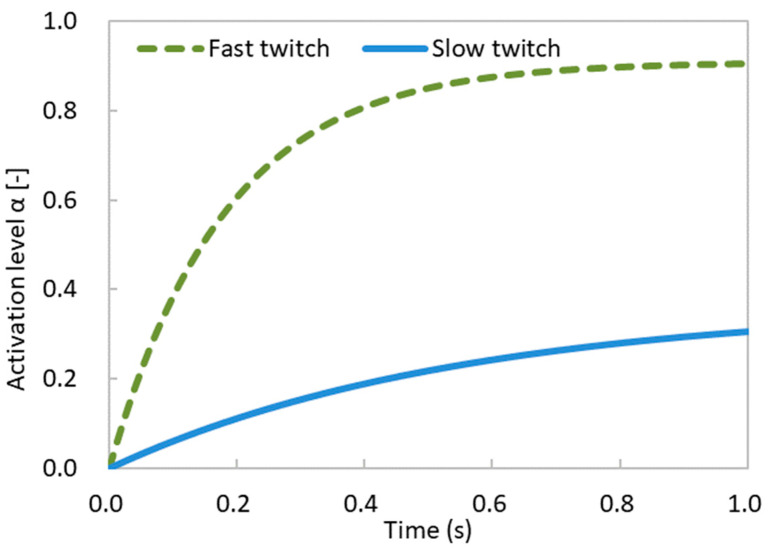
Temporal variations in muscle activation level for fast-twitch and slow-twitch muscle fibers.

In this study, we differentiated between the two categories of muscle fibers in our muscle model by adjusting the values of $\tau_{\mathrm{rise}\;}$ and $\tau_{\mathrm{fall}\;}$ in Equation (10). We set $\tau_{\mathrm{rise}\;}=0.1$ and $\tau_{\mathrm{fall}\;}=1.0$ for fast-twitch muscle fibers, while for slow-twitch muscle fibers, $\tau_{\mathrm{rise}\;}=0.3$ and $\tau_{\mathrm{fall}\;}=3.0$. Neural excitation u=0.5 was used for both types of muscle fibers. The temporal variations in the muscle activation level for fast-twitch and slow-twitch muscle fibers are shown in Figure 8.

Based on Equations (4), (5) and (7), the nominal stress of muscle, including the contributions of both fast-twitch and slow-twitch muscle fibers, is as follows:

(23) $$\begin{array}{ll} T\left({\tilde{\lambda }}_{f},{\dot{\tilde{\lambda }}}_{f},\alpha_{SO},\alpha_{FG}\right) & =T_{SO}\left({\tilde{\lambda }}_{f},{\dot{\tilde{\lambda }}}_{f},\alpha_{SO}\right)+T_{FG}\left({\tilde{\lambda }}_{f},{\dot{\tilde{\lambda }}}_{f},\alpha_{FG}\right)\\ & =T_{o}^{M}\mu_{SO}\left[f_{PE}\left({\tilde{\lambda }}_{f}\right)+f_{L}^{CE}\left({\tilde{\lambda }}_{f}\right)f_{V}^{CE}\left({\tilde{\lambda }}_{f},{\dot{\tilde{\lambda }}}_{f}\right)\alpha_{SO}\right]\\ & +T_{o}^{M}\mu_{FG}\left[f_{PE}\left({\tilde{\lambda }}_{f}\right)+f_{L}^{CE}\left({\tilde{\lambda }}_{f}\right)f_{V}^{CE}\left({\tilde{\lambda }}_{f},{\dot{\tilde{\lambda }}}_{f}\right)\alpha_{FG}\right] \end{array}$$

where $T_{so}$ and $T_{FG}$ represent the muscle stresses generated by slow and fast-twitch muscle fibers, respectively. Furthermore, $\mu_{SO}$ and $\mu_{FG}$ denote the percentages of slow-twitch and fast-twitch muscle fibers within the cross-sectional area, respectively. $\alpha_{SO}$ and $\alpha_{FG}$ represent the activation levels of slow-twitch and fast-twitch muscle fibers (Figure 8), respectively.

Alterations in the proportions of fast-twitch fibers play a pivotal role in the age-related decline of muscle function [20,34,35]. As individuals age, there is a proclivity for an augmentation in the proportion of slow-twitch muscle fibers, concomitant with a diminishing trend in the proportion of fast-twitch muscle fibers [38]. In this study, we employed the biceps brachii muscle fiber composition data of young participants (aged 29 ± 5 years), as quantified by Monemi et al., as a baseline (Young in Table 1) [38]. Referring to Hakkinen et al.’s measurements of muscle fiber composition in elderly individuals (61 ± 4 years) [39], we systematically decreased the percentage of fast-twitch muscle fibers by 7%, taking into account the specific context of aging (Elderly I and II in Table 1). Assuming a linear decline in the percentage of fast-twitch muscle fibers during the aging process, we supposed the young case at 30 (±5 years), Elderly I at 45 (±5 years), and Elderly II at 60 (±5 years).

**Figure 9:**
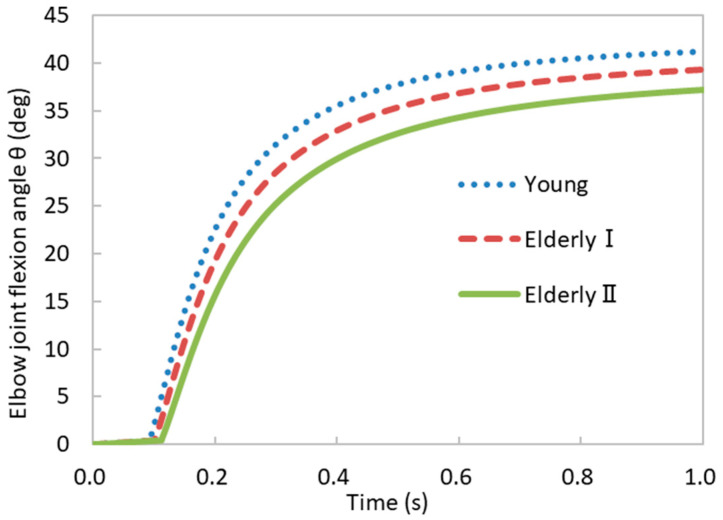
Temporal variations in the elbow joint flexion angle under different muscle fiber compositions.

The simulation results showed an increasing trend in the elbow joint flexion angle over time (Figure 9). An increased percentage of fast-twitch muscle fibers resulted in an elevated elbow joint flexion angle throughout the entire time frame. Additionally, at approximately t = 0.1 s, the initiation of forearm elevation was observed. The sequence of forearm initiation unfolded as follows: Young individuals led the way, followed by Elderly I, and finally, Elderly II. A reduced percentage of fast-twitch muscle fibers led to a delay in the onset of forearm elevation. In instances where fast-twitch muscle fibers were scarce, forearm flexion movements were predominantly governed by slow-twitch muscle fibers, which possess a reduced stress generation capacity. It is noted that this conclusion presupposed the maintenance of all other factors in a constant state. According to Mannion et al. [40], the predominant factor influencing high-intensity exercise capacity is the proportion of fast-twitch muscle fibers. A greater proportion of fast-twitch muscle contributes to a superior dynamic exercise performance. Our findings substantiated the pivotal role of muscle fiber composition in the context of forearm flexion dynamics.

**Figure 10:**
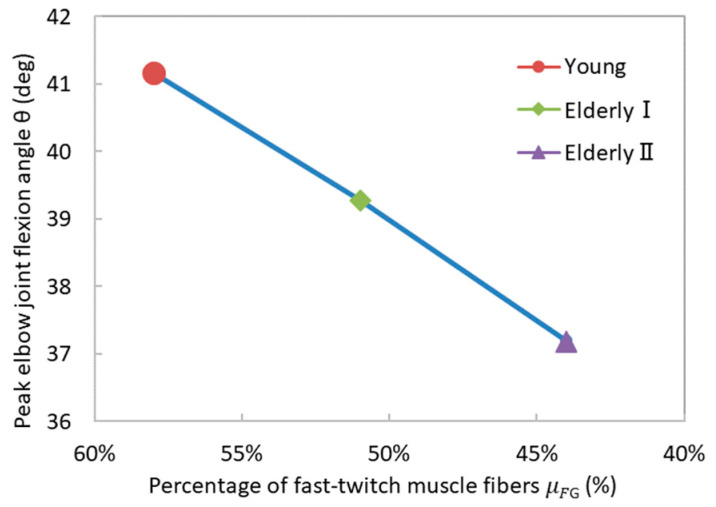
The relationship between the percentage of fast-twitch muscle fibers and peak elbow joint flexion angles.

The maximal degrees of elbow flexion for the Young, Elderly I, and Elderly II cases corresponded to 41.17, 39.28, and 37.19 degrees, respectively (refer to Figure 10). A linear relationship was found between the percentage of fast-twitch muscle fibers and the peak elbow joint flexion angle. As elucidated by Equation (23), there exists a direct linear correspondence between the percentage of fast-twitch muscle fibers and muscle stress. Under the same forearm loading conditions, the greater the proportion of fast-twitch muscle fibers in the biceps brachii, the larger the muscle stress generated, facilitating forearm flexion and resulting in an increased elbow joint flexion angle. The simulation results were consistent with the theoretical model.

We also noted that, with every 10% augmentation in the percentage of fast-twitch muscle fibers, there ensued a corresponding increment of 2.84 degrees in the maximal elbow joint flexion angle. This quantitative analysis introduces a novel methodology for exploring the impact of muscle fiber composition on forearm dynamics. Our findings substantiate the notion that a training program focused on fast-twitch muscle fibers can enhance the forearm flexion ability among older individuals when performing activities under forearm loading conditions. In accordance with Wilson et al. [41], high-intensity, low-volume, and/or high-velocity training programs contribute to an increase in the proportion of fast-twitch muscle fibers. Therefore, engaging in brisk-paced dumbbell bicep curls with lightweight resistance may mitigate the mobility decrease induced by alterations in muscle fiber composition [41,42], aiding in preserving independence and autonomy in the daily lives of elderly individuals.

#### 3.2.2. Biceps Brachii Strength Decline

**Figure 11:**
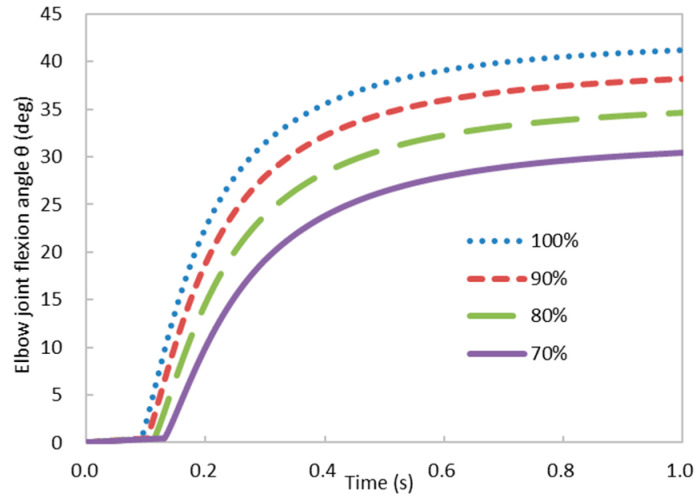
Temporal evolution of the elbow joint flexion angle under varying percentages of maximum muscle stress.

The aging progression also triggers a conspicuous decline in both muscle mass and strength. This decline becomes particularly evident beyond the age of 40. In comparison to young individuals, those surpassing this age threshold encounter a diminution in muscle strength ranging from 16.6% to 40.9% [43,44]. To elucidate the impact of the decline in the biceps brachii strength on forearm flexion, we introduced a reduction in the maximum muscle stress parameter $T_{o}^{M}$ (Equation (23)) from 100% to 70%. The remaining parameters of the simulation remained consistent with those applied to the Young case (refer to Section 3.2.1). The temporal evolution of the elbow joint flexion angle under varying percentages of maximum muscle stress is depicted in Figure 11.

It is noted that an elevated maximum muscle stress $T_{o}^{M}$ led to a greater elbow joint flexion angle throughout the entire temporal duration (Figure 11). Additionally, at approximately  t=0.1 s, the forearm started to elevate. A reduction in the maximum muscle stress triggered a delayed commencement of forearm elevation. This delay is attributed to the diminished maximal muscle stress, resulting in a smaller actual muscle force at an equivalent level of activation (Equation (23)). This reduction necessitated an extended duration to reach the requisite muscle force for the elevation of the load, ultimately introducing a temporal lag in the initiation of forearm elevation.

**Figure 12:**
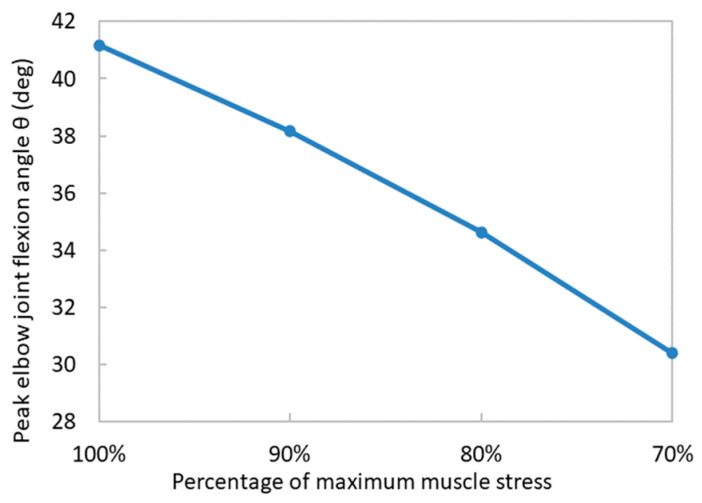
The relationship between the percentage of maximum muscle stress and the peak elbow joint flexion angle.

The elbow joint flexion angles under different maximum muscle stresses are illustrated in Figure 12. As the maximum muscle stress $T_{o}^{M}$ diminished, the peak elbow joint flexion angle also decreased. A nearly linear relationship was observed between the maximum elbow joint flexion angle and the magnitude of the maximum muscle stress. According to Equation (23), the maximum muscle stress parameter acts as a coefficient for generating the muscle force during muscle contraction. Therefore, a greater maximum muscle stress promotes forearm flexion. According to Jung et al. [45], there exists a positive correlation between muscle strength and range of motion. Our simulation results were consistent with this observation.

It was also observed the peak elbow joint flexion angle exhibited a decrement of 3.59 degrees for every 10% reduction in the maximum muscle stress. This quantitative analysis provides a novel perspective for comprehending the impact of diminishing muscle strength on forearm movements. Our study showed that the maximum muscle stress plays an important role in forearm flexion movements. According to Kalapotharakos et al. [46], the continuation of a strength training program is essential for maintaining muscle strength and functional performance in old individuals. Moderate resistance strength training from 2 to 3 days per week can effectively prevent age-related muscle strength decline and improve the quality of life for elderly individuals [47,48].

#### 3.2.3. Neurological Dysfunction

**Figure 13:**
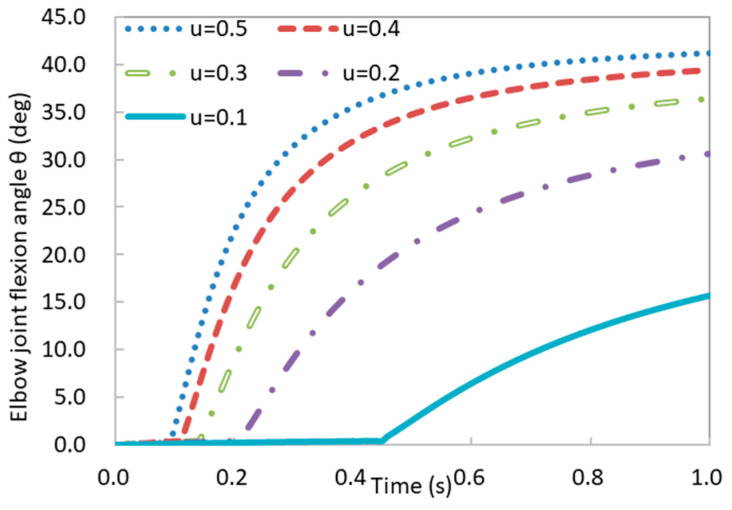
Temporal variations in the elbow joint flexion angle under different neural excitations.

From the generation of neural signals to the initiation of muscle contractions, numerous age-related changes occur in the nervous system. These changes collectively contribute to neurological dysfunction [49,50,51,52,53]. Within the framework of this investigation, we fine-tuned the neural excitation parameter u to emulate the manifestation of neurological dysfunction. It was posited that neural excitation would diminish incrementally from 0.5 to 0.1 in discrete 0.1 intervals, with the aim of replicating the progression of neurological dysfunction. The rest of the simulation conditions were the same as for the Young case (refer to Section 3.2.1). The temporal variations in the forearm flexion angle under different neural excitations are shown in Figure 13.

Our observations indicated a positive relationship between the magnitude of neural excitation and the degree of elbow flexion during forearm movement (Figure 13). At approximately 0.1 s, the forearm endowed with the highest neural excitation level (u=0.5) commenced its elevation. The forearm with the smallest neural excitation (u=0.1) commenced its elevation at 0.42 s. A greater neural excitation level led to an earlier initiation of forearm elevation. Furthermore, we observed that, in comparison to u=0.2, when u=0.1, there was a significant reduction in the elbow joint flexion angle, accompanied by a noticeable postponement in the timing of forearm elevation. This reduction in neural excitation corresponds to diminished muscle activation levels and muscle stresses (Equations (10) and (23)), resulting in a decline in forearm motion capability. As elucidated by Kwon et al. [54], age-related nervous system changes affect the entire process, from neural signal generation to muscle contraction, further impacting motor performance and muscle force. Our simulation results were consistent with the experimental findings.

**Figure 14:**
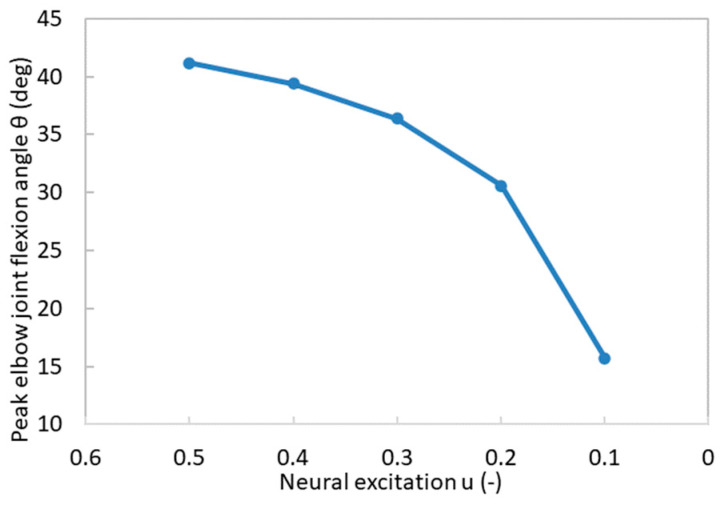
The relationship between peak elbow joint flexion angles and neural excitations.

The relationship between peak elbow joint flexion angle and neural excitation is illustrated in Figure 14. As the neural excitation decreased, a corresponding reduction occurred in the peak elbow joint flexion angle. This relationship is notably nonlinear, due to the fact that, muscle stress, which has a direct relationship with the peak flexion angle, exhibits a nonlinear association with neural excitation (Equations (10) and (23)). Especially when u<0.2, there was a marked decrease in the peak elbow joint flexion angle. These findings suggest that u=0.2 serves as a critical threshold, below which, the forearm’s motion capability undergoes a substantial diminishment. When neural excitation *u* surpasses the 0.2 threshold, each 10% reduction in neural excitation u corresponds to a concomitant decrease of 1.76 degrees in the peak elbow joint flexion angle. Conversely, when u falls below the threshold of 0.2, each 10% reduction in neural excitation leads to a more pronounced decline of 7.44 degrees in the peak elbow joint flexion angle.

Clark et al.’s [49] research indicated that age-related strength decline is not only linked to muscle atrophy, but also depends on neurological factors. Our findings can help to explain the impact of neural excitation on forearm movement under loading conditions. Furthermore, our simulation provided valuable information for determining the critical threshold of neural excitation.

#### 3.2.4. Implication of Our Simulations

We succinctly encapsulated the repercussions of a 10% decrement in the investigated factors on the peak flexion angle (Table 2). In instances where the neural excitation value exceeded the threshold (*u* > 0.2), our findings revealed that the most significant influence on forearm flexion kinetics stemmed from the reduction in the biceps brachii strength, resulting in a reduction of 3.59 degrees. Subsequently, alterations in muscle fiber composition contributed to a decrease of 2.84 degrees, while neurological dysfunction exerted the least impact, with a decline of 1.76 degrees. Conversely, when the neural excitation value fell below the threshold (*u* < 0.2), neurological dysfunction emerged as the paramount determinant affecting forearm flexion movements in older individuals, manifesting a substantial reduction of 7.44 degrees.

When the neural excitation value exceeded the threshold (*u* > 0.2), the decline in the biceps brachii strength became the most significant factor influencing forearm movement, compared to changes in muscle fiber composition. The reason is that the decrease in muscle maximum stress caused by muscle strength decline directly affected the muscle force output (Equation (23)). However, changes in muscle fiber composition indirectly influenced the muscle force and had a smaller impact. When the neural excitation value was under the threshold (*u* < 0.2), the non-linear relationship between neural excitation and the activation level led to a significant decline in muscle stress (Equations (10) and (23)), making it the main factor influencing elbow flexion movement.

According to Clark et al. [49], the aging process is accompanied by a gradual reduction in motor neurons, which impacts the motion capabilities of elderly individuals. When the critical threshold for the number of motor neurons is reached (typically occurring between the ages of 65 and 80), functional disabilities manifest [55]. Our simulation results hold the promise of providing assistance in the development of targeted exercise programs for elderly individuals across different age groups. For elderly individuals under the age of 65, focusing on muscle strength training has proved to be the most effective method for enhancing their motion capabilities, which encompasses implementing progressive strength training or moderate resistance strength training [46]. For those aged 65 and older, exercise programs aimed at improving neurological system functionality become the most effective means for improving their motion capabilities. For instance, developing a training regimen that combines high-resistance training with explosive exercises is an effective approach [56,57].

## 4. Conclusions

In this study, we developed a simplified forearm finite element model including radius, biceps brachii, and tendons to simulate the forearm flexion motion. Since only the biceps brachii muscle was considered in our study, the synergistic interactions among other tissues were neglected. Therefore, the more complicated motions such as the neck behind-arm flexion extension and bench press movement cannot be reproduced by this model. In this work, we also conducted a parametric study to quantitatively investigate the effects of factors including muscle contraction speed, the proportion of fast-twitch fibers, maximum muscle stress, and neural excitation on the flexion angle with a dumbbell load based on the developed model. In the future, a comprehensive experimental investigation to quantitatively measure these parameters will be necessary for validating the accuracy of our simulation results.

From the simulation results produced by this simplified model, we observed that a slow muscle contraction velocity facilitates the forearm flexion movement, which agreed with the trend of the F-V curve. By analyzing the influence exerted by various factors on forearm motion, we also obtained the subsequent findings:

- A larger percentage of fast-twitch muscle fibers and a greater maximum muscle stress value are benefits for forearm flexion movement;
- The fast-twitch muscle fibers’ percentage and maximum muscle stress are linearly correlated with the maximum elbow joint flexion angle;
- The enhancement of neural excitation levels yields advantages in forearm flexion, showcasing a non-linear interplay between neural excitation and the maximum elbow joint flexion angle;
- Moreover, our findings indicated that, when neural excitation levels surpass the threshold, the preeminent factor governing arm flexion movements is the maximal muscle stress. Conversely, in situations where neural excitation levels fall below the threshold, neural excitation takes precedence as the dominant influence on forearm flexion dynamics. Our findings hold significant implications for understanding the mechanisms of forearm motion and guiding rehabilitation training for sarcopenia.

## Figures and Tables

**Table 1:** Muscle fiber compositions of each case.

Case	Young	Elderly I	Elderly II
Fast-twitch [%]	58	51	44
Slow-twitch [%]	42	49	56

**Table 2:** The peak elbow joint flexion angle reduction in elderly subjects under different factors.

	Muscle Fiber Composition Change	Biceps Brachii Strength Decline	Neurological Dysfunction (*u* > 0.2)	Neurological Dysfunction (*u* < 0.2)
Reduced angle (deg)	2.84	3.59	1.76	7.44

**Figure A1:**
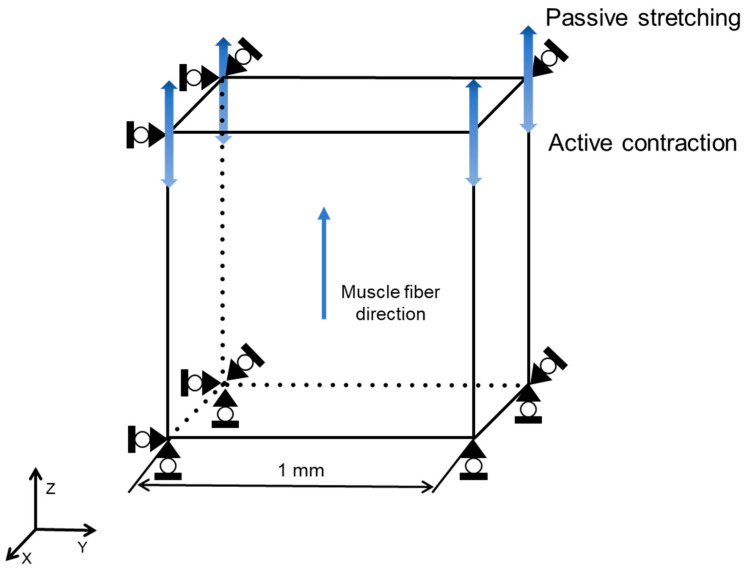
The boundary conditions for the uniaxial passive stretching and active contraction of the muscle model.

**Figure A2:**
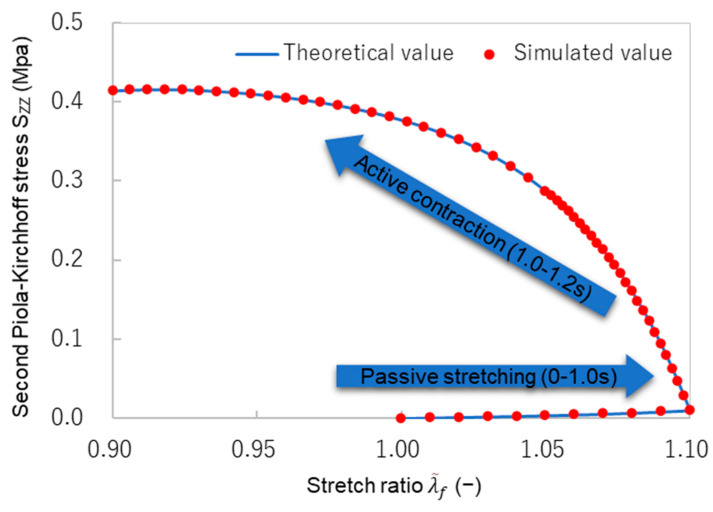
The relationship between the stress ratio ${\tilde{\lambda }}_{f}\;$and the second Piola–Kirchhoff stress $S_{zz}$ during the muscle model’s passive stretching and active contraction.

## Data Availability

The data that support the findings of this study are available from the corresponding author upon reasonable request.

## Supplementary Materials

The following supporting information can be downloaded at: https://www.mdpi.com/article/10.3390/bioengineering11010023/s1, Video S1: Animation of the forearm flexion movement simulated by our finite element model.

## Appendix A. Validation

To validate the muscle finite element model, a single hexahedral element with a 1 mm side length (Figure A1) was used to simulate the muscle stretching in the passive state and contraction in the active state. The muscle fiber direction was aligned along the *Z*-axis. The boundary conditions for uniaxial passive stretching and active contraction of the muscle model are illustrated in Figure A1. Additionally, by applying enforced displacement, the upper four nodes underwent the following conditions: a. they were uniformly stretched along the *Z*-axis at a rate of 0.1 mm/s from 0 to 1 s (passive stretching). b. from 1 to 1.2 s, they experienced uniform contraction at a rate of −1 mm/s (active contraction). In this simulation, we utilized the maximum muscle stress $T_{o}^{M}=0.6688\;$ Mpa. The neural excitation value was set as u=0 during 0–1 s (passive stretching) and u=0.5 during 1–1.2 s (active contraction), while the rest of the simulation parameters were the same as in Section 2.2.

We also computed the theoretical value of the second Piola–Kirchhoff stress $S_{zz}$, as shown in Equation (A1):

(A1) $$\begin{array}{ll} S_{zz} & =-\frac{1}{C_{yy}^{-1}}\left\{cbexp\left(b\left({\tilde{I}}_{C}-3\right)\right)\left(2J^{-\frac{2}{3}}-\frac{2}{3}{\tilde{I}}_{C}C_{yy}^{-1}\right)\right.\\ & +T_{o}^{M}\left\{-\frac{1}{3}{\tilde{\lambda }}_{f}C_{yy}^{-1}\right\}\left\{2Aaexp\left(a{\left({\tilde{\lambda }}_{f}-1\right)}^{2}\right)\left({\tilde{\lambda }}_{f}-1\right)\right.\\ & \left.\left.+\alpha \left[-4{\left({\tilde{\lambda }}_{f}-1\right)}^{2}+1\right]\left[-\frac{1}{\mathrm{atan}\left(5\right)}\mathrm{atan}\left(-0.5{\dot{\tilde{\lambda }}}_{f}\right)+1\right]\right\}\right\}C_{zz}^{-1}\\ & +cbexp\left(b\left({\tilde{I}}_{C}-3\right)\right)\left(2J^{-\frac{2}{3}}-\frac{2}{3}{\tilde{I}}_{C}C_{zz}^{-1}\right)\\ & +T_{o}^{M}\left\{J^{-\frac{2}{3}}{\tilde{\lambda }}_{f}^{-1}-\frac{1}{3}{\tilde{\lambda }}_{f}C_{zz}^{-1}\right\}\left\{2Aae^{a{\left({\tilde{\lambda }}_{f}-1\right)}^{2}}\left({\tilde{\lambda }}_{f}-1\right)\right.\\ & +\left.\alpha \left[-4{\left({\tilde{\lambda }}_{f}-1\right)}^{2}+1\right]\left[-\frac{1}{\mathrm{atan}\left(5\right)}\mathrm{atan}\left(-0.5{\dot{\tilde{\lambda }}}_{f}\right)+1\right]\right\} \end{array}$$

where, under uniaxial passive stretching and active contraction condition, there exist: $C_{yy}^{-1}={\tilde{\lambda }}_{f}$, $C_{zz}^{-1}={\tilde{\lambda }}_{f}^{-2},$ ${\tilde{I}}_{c}={\tilde{\lambda }}_{f}^{2}+2{\tilde{\lambda }}_{f}^{-1},$ ${\dot{\tilde{\lambda }}}_{f}=0.1s^{-1}$ (passive stretching), ${\dot{\tilde{\lambda }}}_{f}=-1s^{-1}$ (active contraction). Additionally, according to Equation (10), during passive stretching α=0, where during active contraction, 0<α≤0.905.

The simulated results in terms of the second Piola–Kirchhoff stress $S_{zz}\;$ are compared with the theoretical values in Figure A2. Figure A2 illustrates the relationship between the $S_{zz}$ and the muscle fiber stretch ratio ${\tilde{\lambda }}_{f}$ during passive stretching and active contraction. The simulated $S_{zz}$ results matched the theoretical curve throughout the entire process, which validated the finite element discretization.

## References

[1] Ambrósio J., Quental C., Pilarczyk B., Folgado J., Monteiro J. (2011). Multibody biomechanical models of the upper limb. Procedia IUTAM.

[2] Teran J., Sifakis E., Blemker S.S., Ng-Thow-Hing V., Lau C., Fedkiw R. (2005). Creating and simulating skeletal muscle from the visible human data set. IEEE Trans. Vis. Comput. Graph..

[3] Dereshgi H.A., Serbest K., Balik B., Sahin S.N. (2022). Stress-strain response of muscle fibers in biceps brachii under dynamic force: An analysis of biceps curl exercise. Politeknik Dergisi.

[4] Dereshgi H.A. (2023). The rest-pause biceps curl exercise effect on biceps brachii muscle of women: A study of mechanical responsiveness. IEEE Access.

[5] Chen D.T., Zeltzer D. Pump It Up: Computer animation of a biomechanically based model of muscle using the finite element method. Proceedings of the 19th Annual Conference on Computer Graphics and Interactive Techniques.

[6] Blemker S.S., Pinsky P.M., Delp S.L. (2005). A 3D model of muscle reveals the causes of nonuniform strains in the biceps brachii. J. Biomech..

[7] Hopkins P.M. (2006). Skeletal muscle physiology. Contin. Educ. Anaesth. Crit. Care Pain.

[8] Greig C.A., Jones D.A. (2013). Muscle physiology and contraction. Surgery.

[9] Zhang G., Chen X., Ohgi J., Miura T., Nakamoto A., Matsumura C., Sugiura S., Hisada T. (2016). Biomechanical simulation of thorax deformation using finite element approach. Biomed. Eng. Online.

[10] Best T.M., McElhaney J.H., Garrett W.E., Myers B.S. (1995). Axial strain measurements in skeletal muscle at various strain rates. J. Biomech. Eng..

[11] Jiang F., Sakuramoto I., Nishida N., Onomoto Y., Ohgi J., Chen X. (2023). The mechanical behavior of bovine spinal cord white matter under various strain rate conditions: Tensile testing and visco-hyperelastic constitutive modeling. Med. Biol. Eng. Comput..

[12] Dao T.T., Tho M.C.H.B. (2018). A systematic review of continuum modeling of skeletal muscles: Current trends, limitations, and recommendations. Appl. Bionics. Biomech..

[13] Wakeling J.M., Febrer-Nafría M., De Groote F. (2023). A review of the efforts to develop muscle and musculoskeletal models for biomechanics in the last 50 years. J. Biomech..

[14] Kojic M., Mijailovic S., Zdravkovic N. (1998). Modelling of muscle behaviour by the finite element method using Hill’s three-element model. Int. J. Numer. Methods. Eng..

[15] Tang C.Y., Tsui C.P., Stojanovic B., Kojic M. (2007). Finite element modelling of skeletal muscles coupled with fatigue. Int. J. Mech. Sci..

[16] Johansson T., Meier P., Blickhan R. (2000). A finite-element model for the mechanical analysis of skeletal muscles. J. Theor. Biol..

[17] Martins J.A.C., Pires E.B., Salvado R., Dinis P.B. (1998). A numerical model of passive and active behavior of skeletal muscles. Comput. Methods Appl. Mech. Eng..

[18] Martins J.A.C., Pato M.P.M., Pires E.B. (2006). A finite element model of skeletal muscles. Virtual Phys. Prototyp..

[19] Tanimoto Y., Watanabe M., Sun W., Tanimoto K., Shishikura K., Sugiura Y., Kusabiraki T., Kono K. (2013). Association of sarcopenia with functional decline in community-dwelling elderly subjects in Japan. Geriatr Gerontol Int..

[20] Marcell T.J. (2003). Sarcopenia: Causes, consequences, and preventions. J. Gerontol. A Biol. Sci. Med. Sci..

[21] Yang J., Jiang F., Yang M., Chen Z. (2022). Sarcopenia and nervous system disorders. J. Neurol..

[22] Netter F.H. (2006). Atlas of Human Anatomy.

[23] Zeng W., Hume D.R., Lu Y., Fitzpatrick C.K., Babcock C., Myers C.A., Rullkoetter P.J., Shelburne K.B. (2023). Modeling of active skeletal muscles: A 3D continuum approach incorporating multiple muscle interactions. Front. Bioeng. Biotechnol..

[24] Humphrey J.D., Yin F.C.P. (1987). On constitutive relations and finite deformations of passive cardiac tissue: I. A pseudostrain-energy function. J. Biomech. Eng..

[25] Pandy M.G., Zajac F.E., Sim E., Levine W.S. (1990). An optimal control model for maximum-height human jumping. J. Biomech..

[26] Bosisio M.R., Talmant M., Skalli W., Laugier P., Mitton D. (2007). Apparent Young’s modulus of human radius using inverse finite-element method. J. Biomech..

[27] Wakabayashi I., Itoi E., Sano H., Shibuya Y., Sashi R., Minagawa H., Kobayashi M. (2003). Mechanical environment of the supraspinatus tendon: A two-dimensional finite element model analysis. J. Shoulder. Elbow. Surg..

[28] Zienkiewicz O.C., Taylor R.L., David D.F. (2013). The Finite Element Method for Solid and Structural Mechanics.

[29] Morrey B.F. (2008). The Elbow and Its Disorders.

[30] Yang J., Lee J., Lee B., Kim S., Shin D., Lee Y., Lee J., Han D., Choi S. (2014). The effects of elbow joint angle changes on elbow flexor and extensor muscle strength and activation. J. Phys. Ther. Sci..

[31] Chow J.W., Darling W.G. (1999). The maximum shortening velocity of muscle should be scaled with activation. J. Appl. Physiol..

[32] Gülch R.W. (1994). Force-velocity relations in human skeletal muscle. Int. J. Sports Med..

[33] Alcazar J., Csapo R., Ara I., Alegre L.M. (2019). On the shape of the force-velocity relationship in skeletal muscles: The linear, the hyperbolic, and the double-hyperbolic. Front. Physiol..

[34] Nilwik R., Snijders T., Leenders M., Groen B.B., van Kranenburg J., Verdijk L.B., Van Loon L.J.C. (2013). The decline in skeletal muscle mass with aging is mainly attributed to a reduction in type II muscle fiber size. Exp. Gerontol..

[35] Walston J.D. (2012). Sarcopenia in older adults. Curr. Opin. Rheumatol..

[36] Eberstein A., Goodgold J. (1968). Slow and fast twitch fibers in human skeletal muscle. Am. J. Physiol..

[37] Simoneau J.A., Bouchard C. (1995). Genetic determinism of fiber type proportion in human skeletal muscle. FASEB J..

[38] Monemi M., Eriksson P.O., Kadi F., Butler-Browne G.S., Thornell L.E. (1999). Opposite changes in myosin heavy chain composition of human masseter and biceps brachii muscles during aging. J. Muscle Res. Cell Motil..

[39] Hakkinen K., Newton R.U., Gordon S.E., Mccormick M., Volek J.S., Nindl B.C., Gotshalk L.A., Campbell W.W., Evans W.J., Hakkinen A. (1998). Changes in muscle morphology, electromyographic activity, and force production characteristics during progressive strength training in young and older men. J. Gerontol. A Biol. Sci. Med. Sci..

[40] Mannion A.F., Jakeman P.M., Willan P.L. (1995). Skeletal muscle buffer value, fibre type distribution and high intensity exercise performance in man. Exp. Physiol..

[41] Wilson J.M., Loenneke J.P., Jo E., Wilson G.J., Zourdos M.C., Kim J.S. (2012). The effects of endurance, strength, and power training on muscle fiber type shifting. J. Strength Cond. Res..

[42] Simoneau J.A., Lortie G., Boulay M.R., Marcotte M., Thibault M.C., Bonchard C. (1985). Human skeletal muscle fiber type alteration with high-intensity intermittent training. Eur. J. Appl. Physiol..

[43] Keller K., Engelhardt M. (2013). Strength and muscle mass loss with aging process. Age and strength loss. Muscles Ligaments Tendons J..

[44] Frontera W.R., Hughes V.A., Fielding R.A., Fiatarone M.A., Evans W.J., Roubenoff R. (2000). Aging of skeletal muscle: A 12-yr longitudinal study. J. Appl. Physiol..

[45] Jung H., Yamasaki M. (2016). Association of lower extremity range of motion and muscle strength with physical performance of community-dwelling older women. J. Physiol. Anthropol..

[46] Kalapotharakos V.I., Smilios I., Parlavatzas A., Tokmakidis S.P. (2007). The effect of moderate resistance strength training and detraining on muscle strength and power in older men. J. Geriatr. Phys. Ther..

[47] Seguin R., Nelson M.E. (2003). The benefits of strength training for older adults. Am. J. Prev. Med..

[48] Ratamess N.A., Alvar B.A., Evetoch T.E., Housh T.J., Ben Kibler W., Kraemer W.J., Triplett N.T. (2009). Progression models in resistance training for healthy adults. Med. Sci. Sports. Exerc..

[49] Clark D.J., Fielding R.A. (2012). Neuromuscular contributions to age-related weakness. J. Gerontol. A Biol. Sci. Med. Sci..

[50] Jang Y.C., Van Remmen H. (2011). Age-associated alterations of the neuromuscular junction. Exp. Gerontol..

[51] Clark L.A., Manini T.M., Wages N.P., Simon J.E., Russ D.W., Clark B.C. (2021). Reduced neural excitability and activation contribute to clinically meaningful weakness in older adults. J. Gerontol. A Biol. Sci..

[52] Delbono O. (2003). Neural control of aging skeletal muscle. Aging Cell.

[53] Jensen G.L. (2008). Inflammation: Roles in aging and sarcopenia. JPEN J. Parenter. Enteral. Nutr..

[54] Kwon Y.N., Yoon S.S. (2017). Sarcopenia: Neurological point of view. J. Bone. Metab..

[55] McNeil C.J., Doherty T.J., Stashuk D.W., Rice C.L. (2005). Motor unit number estimates in the tibialis anterior muscle of young, old, and very old men. Muscle Nerve.

[56] Häkkinen K., Kallinen M., Linnamo V., Pastinen U.M., Newton R.U., Kraemer W.J. (1996). Neuromuscular adaptations during bilateral versus unilateral strength training in middle-aged and elderly men and women. Acta Physiol. Scand..

[57] Häkkinen K., Kallinen M., Izquierdo M., Jokelainen K., Lassila H., Mälkiä E., Kraemer W.J., Newton R.U., Alen M. (1998). Changes in agonist-antagonist EMG, muscle CSA, and force during strength training in middle-aged and older people. J. Appl. Physiol..

